# Comparison of codon usage in mycobacteriophages with and without tRNAs in their genomes

**DOI:** 10.1186/1471-2105-14-S17-A9

**Published:** 2013-10-22

**Authors:** Ethan S Gill, Claire A Rinehart

**Affiliations:** 1 Department of Computer Science, Western Kentucky University, Bowling Green, KY, 42101, USA; 2 Department of Biology, Western Kentucky University, Bowling Green, KY, 42101, USA

## Background

*Mycobacterium smegmatis* is a soil bacterium. Over 448 mycobacteriophages specific for *M. smegmatis* have been sequenced and grouped into clusters of related genomes based on the similarity of their products and genome organization [1]. Eighty-one of these sequenced phage genomes contain at least one tRNA.

## Materials and methods

**Figure 1:**
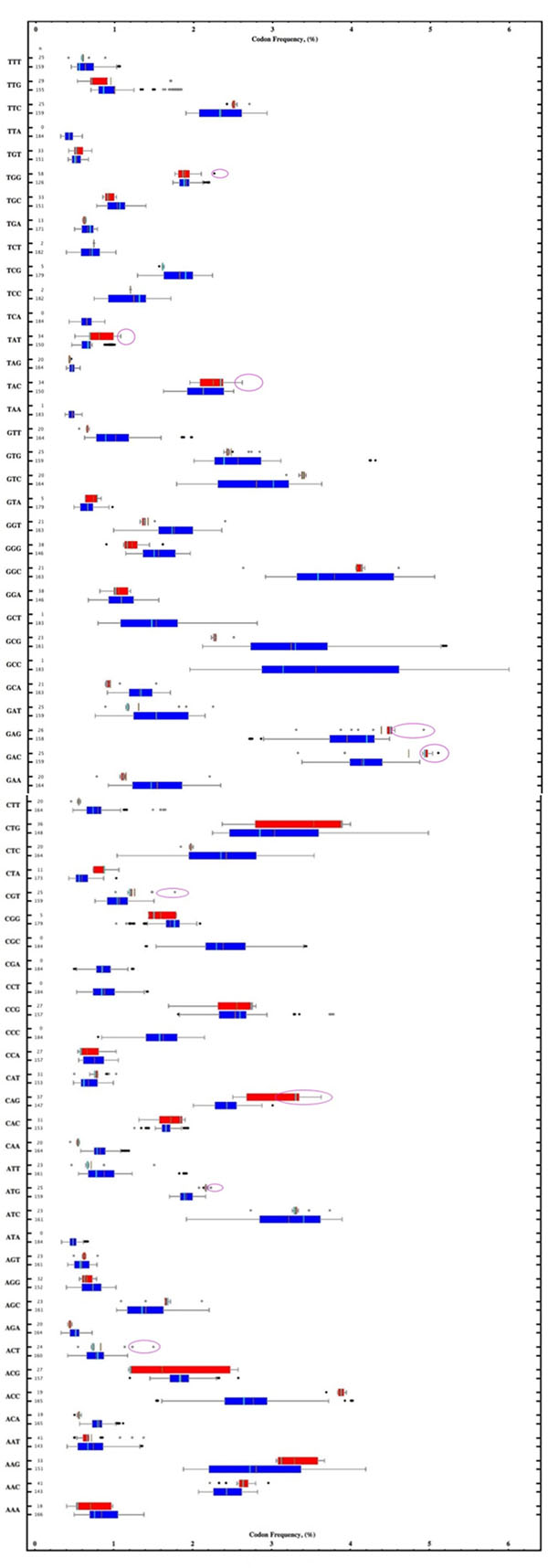
BoxWhiskerChart of codon frequencies in phage without tRNA (blue boxes) and those with tRNA in their genomes (red boxes). Colored boxes encompass the 25%-75% quantile while the whiskers encompass the 0-25% and 75-100% quantiles. Dark dots are outliers while gray dots are far outliers. The brown line is the mean and the cyan line is the median. The numbers on the left margin represent the number of observations, n.

In this study we compared the codon frequencies between the phages that carry tRNA in their genome (Clusters A2, A3, A5, A6, A9, C1, C2, E, J, K1, L1, L2, M) to those phages without tRNA in their genome (Clusters A1, A4, B1, B2, D, F1, G) in order to determine whether the tRNAs being carried by the mycobacteriophages were needed to survive in *M. smegmatis.* The phages with embedded tRNAs were drawn from http://phagesdb.org/data/. The coding sequences for the mycobacteriophages were obtained from NCBI GenBank [2]. Codon frequencies were derived for each mycobacteriophage and a BoxWhiskerChart (Figure 1) was made using a program written in Mathematica^®^.

To determine which codons may require expression of phage tRNAs, Figure 1 plot regions for phages containing tRNA (red) that show frequencies in excess of those not containing tRNA (blue) have been circled in magenta. Only a few codons meet this criteria: ACT, ATG, CAG***, CGT, GAC**, GAG*, TAC, TAT, TGG. A significant number of mycobacteriophages lay outside the non-tRNA phage boundaries for codons CAG, GAC and GAG, therefore, these phages may be dependent on internally coded tRNAs.
